# Speech perception in cochlear implant users with the HiRes 120 strategy: a systematic review

**DOI:** 10.1590/S1808-86942012000300021

**Published:** 2015-10-14

**Authors:** Tatiana Mendes de Melo, Maria Cecília Bevilacqua, Orozimbo Alves Costa

**Affiliations:** a MSc in Speech and Hearing Therapy (Professor of Speech and Hearing Therapy – University of Guarulhos).; b Full Professor at the University of São Paulo – Bauru Campus. Speech and Hearing Therapist. Coordinator at the Audiological Research Center of the Craniofacial Anomalies Rehabilitation Hospital – USP - Campus Bauru.; c Full Professor of Speech and Hearing Therapy – Dental School of Bauru – University of São Paulo. University of São Paulo.

**Keywords:** cochlear implants, hearing loss, speech perception

## Abstract

Despite technological advances employed in signal processing strategies, one of the remaining obstacles are spectral gap details on the information transmitted. Considering its importance in speech perception, researchers have investigated mechanisms to optimize spectral details through virtual spectral channels. The clinical application of this technique resulted in a new approach to signal processing - the HiRes 120.

**Objective:**

To assess the auditory performance of cochlear implant users with the HiRes 120 strategy.

**Methodology:**

The literature review was conducted in an electronic database, with standard bibliographic search in the year 2011, using specific keywords. In order to select and evaluate the scientific studies found in the search, we setup search containing the following aspects: type of study, subjects, intervention used and evaluation of the results.

**Conclusion:**

Scientific evidence points to an improvement in hearing performance in noisy environments with the HiRes 120 strategy, but this does not occur in quiet situations. The optimization of speech perception with this strategy is closely related to the cochlear implant user's age, to the time of sensory deprivation and the acclimatization time required to use the strategy's spectral information.

## INTRODUCTION

The multichannel cochlear implant (CI) represents the most important progress in the treatment of severe to profound bilateral hearing loss in people who do not benefit from an individual sound amplification devices (ISAD).

Signal processing strategies are characterized by a set of rules to convert acoustic signals into electrical stimulation. The input sound is divided into a number of predetermined filters. Each filter will be directed to a specific channel to select the main acoustic characteristics of the input sound, which will be coded into an electrical stimulation.

Despite the technological progresses enjoyed by signal processing strategies, it is still a challenge to provide the conversion of acoustic signals into electrical stimulation with the greatest possible fidelity, in the three dimensions of sound: intensity, time (temporal resolution) and frequency.

One of the still existing obstacles is the gap of spectral details in the transmitted electrical information. In CI, the bundle of electrodes uses cochlear tonotopic characteristics, that is, the high frequency spectral information sent to basal electrodes, while the low frequency information is sent to more apical electrodes, thus enabling different pitches to be perceived by the patient. Nonetheless, the number of spectral information is limited to the number of intracochlear electrodes, which define the number of stimulation spectral bands. Considering the importance of the spectral resolution in speech perception for CI users, researchers investigated mechanisms used to optimize the spectral detail without adding additional electrodes to the bundle inserted in the cochlea, by means of the electrical current guiding – also called virtual spectral channels.

The concept of virtual channels is associated to the stimulation of two adjacent electrodes simultaneously in order to generate perceptions of intermediate pitches, that is, a variation of the proportion of electrical current which is supplied in the two physical contacts resulting in an electrical field, which is guided to the existing neural fibers between the adjacent electrodes, thus creating the virtual spectral channels.

The clinical application of the virtual spectral channels resulted in a new implant signal processing strategy from Advanced Bionics - a HiResolution with Fidelity (HiRes 120), launched in 2006. Although the most accurate representation of the spectral aspect becomes possible with the HiRes 120, it is still not totally clear whether this additional spectral information is significant for the patients. It is important to stress that this is the conception of this new coding strategies, but rather the stimulation of neural structures from different auditory impairment etiologies bringing about different results.

Within this concept, the goal of the present study was to assess the auditory performance of CI users with the HiRes 120 strategy, by means of a systematic review of the literature.

## METHODOLOGY

Considering that the systematic review is done after formulating the specific questions which guide the search of publications, the investigation question of the present study was: “Is there any benefit from the HiRes 120 signal processing strategy, compared to HiRes, in the auditory performance of patients with CI?

**Chart 1:**
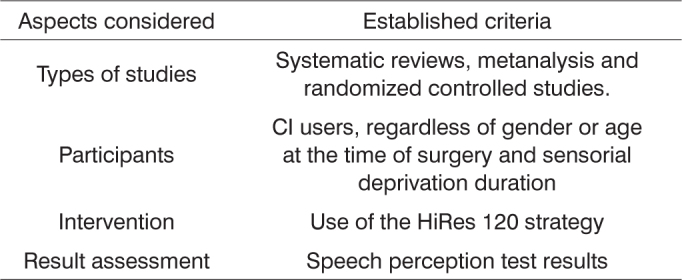
Criteria established for the selection and assessment of the scientific studies found in the literature.

In order to select and assess the scientific studies found in the electronic search, we used criteria involving the following aspects: type of study, participants, intervention used and assessment of results, which can be seen in Chart 1.

**Chart 2:**
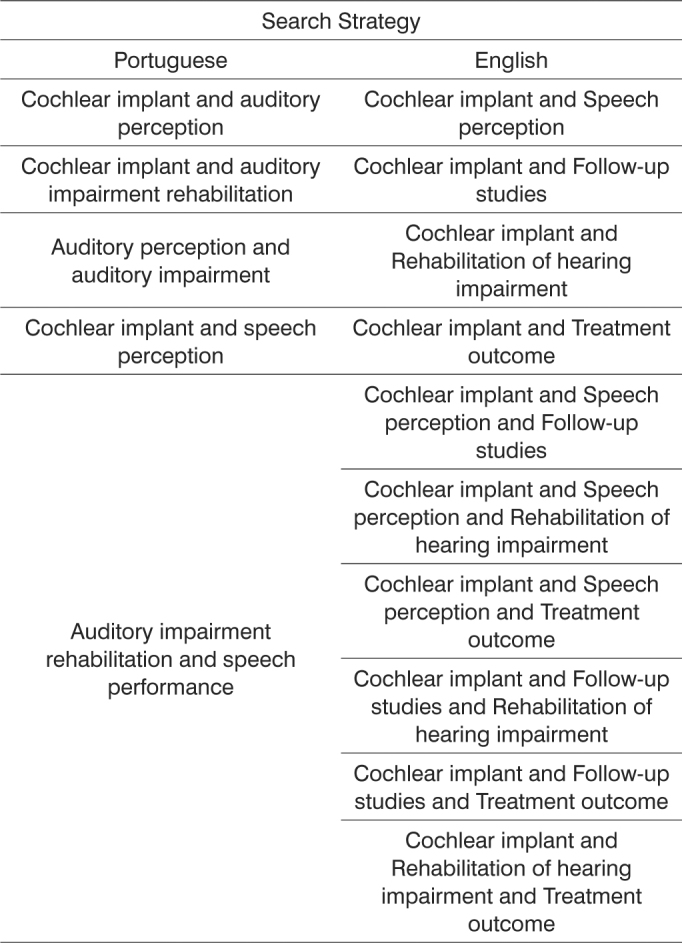
Search strategies used in the literature search.

The bibliographic search used the strategies presented on Chart 2.

The bibliographic search was carried out in publicly available databases, such as: Lilacs, Medline and Scielo; and in restricted access databases, such as: Scorpus, Embase, Science Direct Online and Web of Science. There were no restrictions as to the year of publication, in other words, we analyzed the studies published until March of 2011. After this date, we used the “alert” option, provided by the electronic databases, which is a tool that enables the researcher to receive weekly or monthly information, according to choice, about the publication of new studies associated with the search strategy used in the data base.

In the search we assessed and selected only those papers which title, summary or body had some relationship with the goals of the present study. After selecting the summaries of the papers found, pertaining to the question at hand, the full papers were retrieved. The data from each of the potential relevant papers for the systematic review were collected using a protocol form.

At the end of the search, we selected 38 abstracts; however, we discarded duplicated papers and, then, we ended up with nine potentially relevant studies to be included in the systematic review, which were entirely read.

After reading and analyzing the papers found, six were selected for the systematic review, one was taken off because it tested a HiRes 120 strategy study version, called SpecRes, and six other studies were taken off because of the methodological design, in other words, they were cross-sectional. The flowchart below (Flowchart 1) shows a summary of the process used to obtain the papers selected for the literature systematic review.

**Flowchart 1:**
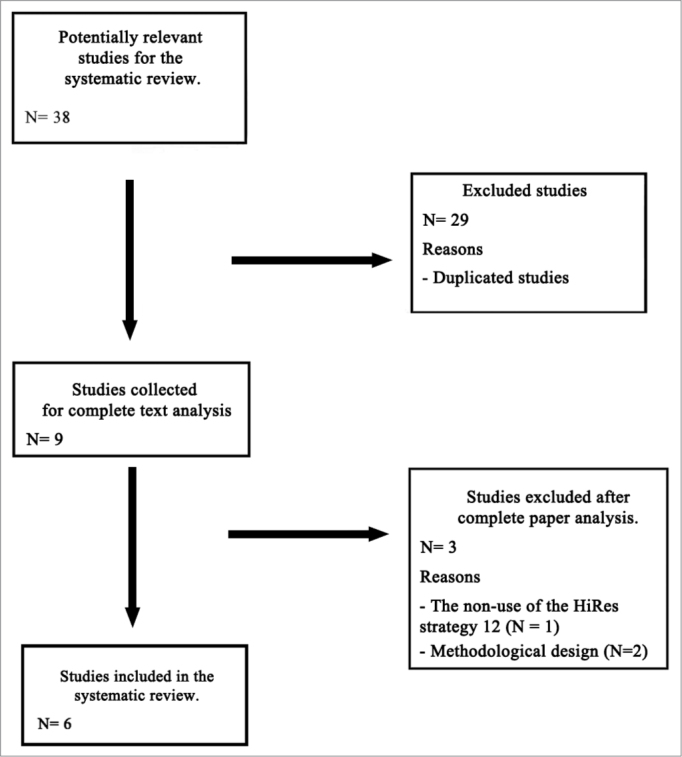
Summary of the process used to obtain the papers selected for the systematic review.

## RESULTS AND DISCUSSION

Cochlear implants (CI) have been dramatically improved along the years, since the development of the single-channel devices from the early 70's. Changes in the electronic quality of the electrode bundle is one of the most important aspects, which together with improvements in sound coding strategies have contributed to an improvement in speech perception for CI users.

The devices from Advanced Bionics started to be commercialized in the early 90's – the Clarion 1.0 and 1.2 devices, with the continuous interleaved strategy (CIS), multiple pulsatile sampler (MPS) and simultaneous analog stimulation (SAS) speech strategies. One major step forward for the Advanced Bionics devices was the introduction of the high resolution (HiRes) speech signal processing, in 2002, which provided a better temporal detailing of the acoustic information than the signal processing strategies previously described. The HiRes works with 16 active channels, with a minimum of 10.8 µs of pulse width and stimulation rate up to 5.156 pps per channel.

Nonetheless, one of the CI limitations is the gap of spectral details present in the electrical information transmitted. This results from a number of factors, such as the irregular survival of the auditory nerve fibers in the specific place where the electrode is stimulating and the limited number of stimulating electrodes in the electrode bundle. Technologically speaking, in 2006, it was possible to employ the virtual spectral channels in a signal processing strategy - HiRes120, thus increasing the intracochlear stimulation sites and, consequently, providing further spectral information for CI users, together with the other benefits already provided with the HiRes strategy.

After consulting the national and international literature in electronic data bases, it was possible to find that the studies which investigated the auditory performance of CI users with the HiRes 120 strategy started to be published in 2008. Of the nine fully revised studies, three were taken off the systematic review – one for testing a version of the HiRes 120^1^ strategy research version called SpecRes and two other studies were taken off because of the methodological design presented^2^, ^3^, that is, studies which made a speech perception longitudinal assessment in CI users after conversion from the HiRes to HiRes 120 strategy; nonetheless, they did not have a control group and did not reassess the auditory performance of the participants with the HiRes strategy after using the HiRes 120. This made unfeasible the maintenance of these systematic review studies, since their results could have been influenced by the learning effect, in other words, performance improvement did not happen thanks to the strategy change, but rather by the entire auditory perception, based on a greater auditory experience with the CI.

Of the six studies included in this systematic review, one involved children with prelingual auditory loss^4^ and five were done with post-lingual hearing loss adults^5^, ^6^, ^7^, ^8^, ^9^. Implementing the HiRes 120 strategy in the pediatric population is more recent in comparison with studies carried out in the adult population, and leads to great expectations, since the additional spectral information provided by the strategy must be better used in cases of short auditory deprivation period and greater residual neural plasticity^4^.

Upon considering the auditory performance of CI users with the HiRes 120 strategy, of the six texts analyzed, all involved the participants' performances in silence and in the presence of competitive noise. Today, speech performance in noise tests are fundamentally important to assess the benefits of using a CI, since it is a measure used to assess auditory demand in situations similar to the ones experienced by the CI user in real life, in which the low signal redundancy impairs speech perception.

Among the results obtained from the analysis of the scientific studies included in the systematic review, two studies showed performance improvements in silence, which were statistically significant^6^, ^7^. Nonetheless, there is a limitation in the studies when pointing to scientific evidence of better auditory performance in users of the HiRes 120 strategy in silent environments because of the “roof effect”, in other words, in baseline evaluations – in which the subjects are assessed with the HiRes strategy, the speech perception results are already near 90% of correct answers and, for that, the change in signal processing strategy does not point to, from the statistical viewpoint, an improvement in auditory perception in this situation^8^.

Moreover, of the six studies analyzed, four proved an improvement in the competitive noise situations with the HiRes 120^4^, ^6^, ^7^, ^8^ strategy. Two studies analyzed did not show auditory processing improvements with the HiRes 120 strategy, both in silence and under noise^5^, ^9^.

Upon analyzing speech perception tests used in the different studies, we see a number of tests utilized to assess the auditory perception with the HiRes 120 strategy, using the Kowal Bamford - BKB-SIN^5^ test, the Hearing in Noise - HINT^6^, ^7^ test, the Speech Sound Evaluation^4^ test, the HSM sentences^8^ test and dissyllable and monosyllable words^9^. This heterogeneity in speech perception tests used to assess the results may influence in their analyses, since the type of noise employed and the signal/noise ratio utilized may have an influence according to the study findings^8^.

Based on the results of the analyzed studies, we noticed that it is possible that the speech perception performance with the HiRes 120 strategy is closely related to the CI user's age^4^, ^5^, with the sensorial deprivation time^4^ and the necessary acclimatization period for the auditory system of each individual to be able to reorganize and make a better use of the spectral information provided by the strategy^5^, ^8^.

Moreover, it is known that in CI results there are numerous factors involved, such as the individual characteristics of CI users, bio-psychosocial aspects, patient and/or family commitment concerning the treatment, specialized speech and hearing therapy and the very hearing loss etiology, and such factors must be investigated in order to assess their contribution to the auditory performance using this speech coding strategy.

## FINAL REMARKS

The scientific evidence of the studies analyzed point to an improvement in the auditory performance under noise with the HiRes 120 strategy. It was not possible to state that the strategy also benefits CI users under silence, because of the “roof effect” influence.

Speech performance optimization using the HiRes 120 is closely associated with the age of the CI user, with the time of sensorial deprivation and the necessary adaptation time for the auditory system to use the spectral information provided by the strategy.

## References

[1] Nogueira W, Litvak L, Edler B, Ostermann J, Buchner A (2009). Signal processing strategies for cochlear implants using current steering. EURASIP J Adv Signal Process.

[2] Chang YT, Yang HM, Lin YH, Liu SH, Wu JL (2009). Tone Discrimination and speech perception benefit in Mandarin-speaking children fit with HiRes fidelity 120 sound processing. Otol Neurol..

[3] Han D, Liu B, Zhou N, Chen X, Kong Y, Liu H (2009). Lexical tone perception with HiResolution and HiResolution 120 sound-processing strategies in pediatric Mandarin-speaking cochlear implant users. Ear Hear..

[4] Mancini P, Bosco E, D'agosta L, Traisci G, Nicastri M, Capelli G (2009). Implementation of perceptual channels in children implanted with a HiRes 90K device. Acta Otolaryngol..

[5] Donaldson GS, Dawson PK, Borden LZ (2011). Within-subjects comparison of the HiRes and Fidelity120 speech processing strategies: speech perception and its relation to place-pitch sensitivy. Ear Hear..

[6] Park HJ, Lee SC, Chun YM, Lee JY (2009). HiRes with Fidelity 120 benefit in native speakers of Korean. Cochlear Impl Int..

[7] Firszt JB, Holden LK, Reeder RM, Skinner MW (2009). Speech recognition in cochlear implant recipients: comparison of standard HiRes and HiRes 120 sound processing. Otol Neurol..

[8] Brendel M, Buechner A, Krueger B, Frohne-Buechner C, Lenarz T (2008). Evaluation of the Harmony soundprocessor in combination with the speech coding strategy HiRes 120. Otol Neurol..

[9] Drennan WR, Won JH, Nie K, Jameyson E, Rubinstein JT (2010). Sensitivity of psychophysical measures to signal processor modification in cochlear implant users. Hear Res..

